# Deep Reinforcement Learning with Explicit Spatio-Sequential Encoding Network for Coronary Ostia Identification in CT Images

**DOI:** 10.3390/s21186187

**Published:** 2021-09-15

**Authors:** Yeonggul Jang, Byunghwan Jeon

**Affiliations:** 1 Graduate School of Medical Science, Brain Korea 21 Project, Yonsei University College of Medicine, Seoul 03722, Korea; ygjang1722@yonsei.ac.kr; 2 School of Computer Science, Kyungil University, Gyeongsan 38428, Korea

**Keywords:** reinforcement learning, localization, coronary computed tomography angiography, coronary ostia

## Abstract

Accurate identification of the coronary ostia from 3D coronary computed tomography angiography (CCTA) is a essential prerequisite step for automatically tracking and segmenting three main coronary arteries. In this paper, we propose a novel deep reinforcement learning (DRL) framework to localize the two coronary ostia from 3D CCTA. An optimal action policy is determined using a fully explicit spatial-sequential encoding policy network applying 2.5D Markovian states with three past histories. The proposed network is trained using a dueling DRL framework on the CAT08 dataset. The experiment results show that our method is more efficient and accurate than the other methods. blueFloating-point operations (FLOPs) are calculated to measure computational efficiency. The result shows that there are 2.5M FLOPs on the proposed method, which is about 10 times smaller value than 3D box-based methods. In terms of accuracy, the proposed method shows that 2.22 ± 1.12 mm and 1.94 ± 0.83 errors on the left and right coronary ostia, respectively. The proposed method can be applied to the tasks to identify other target objects by changing the target locations in the ground truth data. Further, the proposed method can be utilized as a pre-processing method for coronary artery tracking methods.

## 1. Introduction

A landmark identification task is a prerequisite process for medical image analysis. Because a medical image itself has an arbitrary local coordinate system irrelevant to the anatomy, a reformation of the initial coordinate into the new coordinate, based on the patient-specific anatomical landmarks, provides consistent observations for segmentation and registration tasks for medical image analysis. Because the morphometric relationships of human organs in medical images are apparent, landmark identification allows this advantage to be exploited.

**Figure 1:**
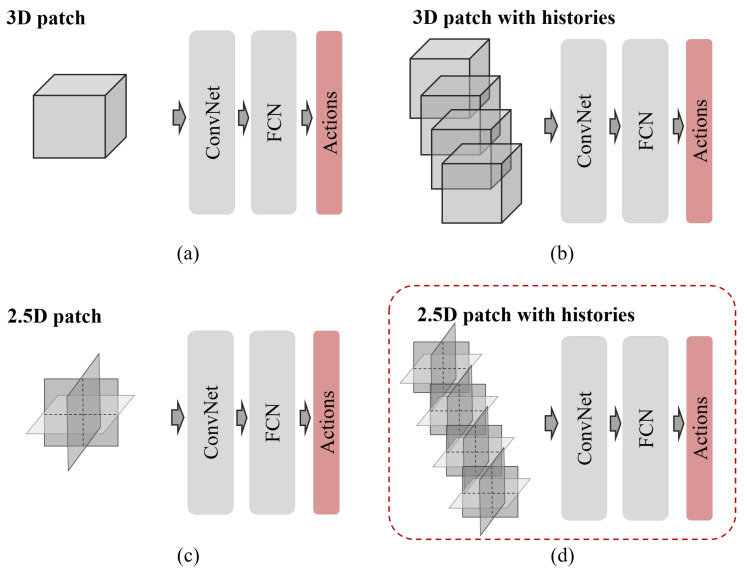
Various state representations: (**a**) single 3D boxed patch, (**b**) 3D patch with multiple histories, (**c**) 2.5D patch with three orthogonal view aggregation, and (**d**) 2.5D patch with multiple histories, with the state used in this study; ConvNet, convolutional neural network; FCN, fully connected network.

Many applications of deep reinforcement learning (DRL) systems have been introduced in the medical field to solve the practical problems of image segmentation, landmark detection, registration, and view planning on three-dimensional (3D) images [1,2,3]. DRL can also be applied to trace blood vessels or to outline anatomical organs [4,5]. Local patch-based observations have mainly been used in 3D volumetric imaging such as computed tomography (CT) or magnetic resonance (MR), as shown in Figure 1. For learning in a 3D space, 2.5D patch-based approaches [6,7,8] and 3D patch-based networks [1,9,10] have been developed. A DRL system with a 3D patch + three past histories, as similarly shown in Figure 1b, has been proposed [2]. Historical information is assumed to stabilize the search trajectories and prevent an agent from becoming stuck in repeated cycles [2,10,11,12].

In DRL implementation, it is necessary to obtain contextual information from the agent’s location to a certain range to observe its current state. However, learning the optimal behavior with a large state is challenging and requires numerous trials and errors in RL problems. There is a trade-off between a 2.5D observation patch and a 3D observation patch in efficiency and the amount of contextual information. It is not yet known which is better: 2.5D or 3D observation patches.

Walid et al. [13] proposed a partial policy-based RL framework that trains multiple policies for each axis (*x, y, z*). Multiple pseudo-3D-based (2n+1 adjacent 2D slices) policies have independent behaviors and are combined with vector additions for each partial action to decide an actual 3D displacement vector, with an attempt to minimize the state size while maintaining the performance. However, 2D adjacent slice modeling uses limited information in the directions of other axes (sagittal and coronal), unlike the axial plane.

In this paper, we propose a novel deep reinforcement learning (DRL) framework based on 2.5D historical observation patches for identifying the left and right coronary artery ostia (LCA and RCA) from 3D CCTA, one of the essential prerequisite tasks for automatic analysis of cardiovascular disease. For modeling the state features from 2.5D historical observation patches, an explicit spatial-sequential encoding policy network (EsseNet) is newly proposed, as shown in Figure 1d. An optimal action policy is determined by explicitly encoding spatio-sequential information from four 2.5D historical observation patches (one current state plus three past histories). Consideration of the historical observation patches enables the agent to learn its orientation and stabilize the search trajectory. The proposed network minimizes the size of the input parameters and accumulates more instances of experience into the experience replay memory than 3D patches in a deep Q-network implementation and thus benefits the DRL system. To the best of our knowledge, this is the first study that proposes a highly optimized network for 2.5D historical observation patches in 3D volumetric medical imaging. In addition, we propose a localization strategy for the agent to reach the target location quickly and accurately by taking a displacement action with variable step sizes in a coarse-to-fine manner. The proposed network is trained with a dueling deep Q-network (Duel DQN) framework on the CAT08 dataset. The experiment results show that our method is more efficient and accurate than conventional approaches. Further, the proposed method can be applied to identify other target objects by changing the target locations in the ground truth data.

## 2. Related Work

There have been attempts to solve the landmark detection problem from several perspectives. Some find solutions by modeling anatomical relations using probabilistic inference [14,15]. The typical machine learning-based approaches effectively use a large-scale annotated dataset [16,17,18]. In addition, collaborating multi-agent based methods were recently introduced for the task of detecting multiple landmarks. Leroy et al. [12] proposed communicative DRL agents for landmark detection in brain images. The agents in the method learn explicit communication channels and implicit communication signals. The method outperforms single and multi-agents approaches for detecting the Brain landmarks. Kasseroller et al. [19] recently proposed a collaborative multi-agent reinforcement learning algorithm for landmark localization using continuous action space. Using a continuous action space reduces the number of steps compared to the other DRL methods based on discrete actions. These methods rely on precise feature engineering for the appearance model because learning it and searching the active objects are conducted independently.

Deep neural networks are known to learn the features automaticallywith better data disentangling pabilities [20]. Amir et al. [2] proposed and evaluated a suite of Deep Q-Network (DQN) based approaches to identify several cardiac and brain landmarks from multimodal images. With the method, 3D patches with three histories are considered for the state of the agent. Ghesu et al. [1] proposed a multi-scale DQN to detect 3D landmarks in CT scans and used a single boxed region of interest (ROI) for the state.

There is a trade-off between the exploitation of more contextual information for feature extraction and more accumulation for generalization. By using 2.5D patches, the number of operations can be reduced by approximately half compared to 3D patches. When fetching random batches from experience and replay memory, it can be composed with as much data as possible, improving the generalization. From this perspective, using 2.5D patches in DQN implementations might be advantageous because more experience data can be accumulated in the experience replay memory than with 3D patches.

As a decomposition representation, a 2.5D CNN is an alternative learning approach to reduce the computational cost compared to 3D patch-based learning tasks [6]. It uses input data consisting of three orthogonal planes (axial, coronal, and sagittal planes) as channels. Despite the achievement of superior performance in 3D medical applications [7,8], planes from different axes have less local proximity to each other as they get farther from the center, and thus combining them as a channel may be insufficient. Because three planes contain different information, it is more appropriate to separate convolutional layers for each axis to encode the features [21].

## 3. Background

To apply the proposed network, we chose a deep Q-learning-based method similar to the way that humans remember and learn through trial and error. Unlike other reinforcement learning methods, because the proposed approach utilizes the experience and replay memory, it is necessary to reduce the size of the experience instance by using the optimal size of the observation information needed to select the optimal action. We will briefly review the theory of a DQN in the following subsections.

### 3.1. Q-Learning

Watkins and Dayan [22] presented and proved a convergence theorem for Q-learning, a simple way for agents to learn to select an optimal action in controlled Markovian domains. The optimal action-selection policy can be found by the learning quality function Q(s,a), which measures the quality of a certain action $a_{t}$ given state $s_{t}$. In addition, Q(s,a) is defined as the expected value of the discounted future rewards $E[r_{t+1}+\gamma r_{t+2}+\ldots +\gamma^{n-1}r_{t+n}]$ where γ is a discount factor. This function can be found recursively based on the Bellman equation [23] as $Q_{i+1}=E\left[r+\gamma \max_{a^{\prime }}Q_{i}\left(s^{\prime },a^{\prime }\right)\right]$, where $s^{\prime }$ and $a^{\prime }$ are the next state and action. The optimal action-selection policy is then found using the highest long-term reward $Q^{*}\left(s,a\right)$.

### 3.2. Deep Q-Learning

Based on Q-learning, briefly described in Section 3.1, a deep neural network with the network parameter θ is an alternative to a table-based policy as Q(s,a)≈Q(s,a;θ). However, reinforcement learning using a non-linear approximator for the Q-function is known to be unstable [24]. The effort required to solve two main causes of instability brought about a breakthrough in the stability of deep reinforcement learning [11]. The main ideas here are to remove the correlations between the adjacent data to make the target data stationary. First, the experience replay buffer is maintained as randomized over the experience data to remove the correlations in the observation sequence. Second, prediction and target models are separated, and the target values are only periodically updated. The deep neural network-based optimal policy is trained using the Bellman optimal equation and the loss function as follows:

(1) $$L_{DQN}\left(\theta \right)=E\left[{\left\{r+\gamma max_{a^{\prime }}Q\left(s^{\prime },a^{\prime };\theta^{-}\right)-Q\left(s,a;\theta \right)\right\}}^{2}\right].$$

where θ and $\theta^{-}$ are parameters for the prediction and target models, respectively.

### 3.3. Dueling Deep Q-Learning

The dueling network architecture [25] is explicitly decomposed into state values and state-dependent action advantages. Here, Q(s,a) is a quality function of a certain action *a* given a certain state *s*.

(2) Q(s,a;θ,α,β)=V(s;θ,β)+A(s,a;θ,α)

where *V* and *A* are the state value and action advantage function, respectively; θ represents the parameters of the convolution layer; and α and β represent the parameters of two fully connected layers for *V* and *A*.

However, Q(s,a;θ,α,β) in Equation (2) is only a parameterized estimate of the true quality function, and *V* and *A* cannot be uniquely identified. The alternative action advantage module in Equation (3) increases the stability of the optimization. The advantages only need to change as quickly as the mean, instead of having to compensate any changes to the advantage of the optimal action in Equation (2).

(3) $$Q\left(s,a;\theta ,\alpha ,\beta \right)=V\left(s;\theta ,\beta \right)+(A\left(s,a;\theta ,\alpha \right)-\frac{1}{\left|A\right|}\sum_{a^{\prime }}A\left(s,a^{\prime };\theta ,\alpha \right))$$

## 4. Automatic Landmark Detection System

### 4.1. Markov Decision Process

The proposed system sequentially searches and fixes 3D locations from a randomly given seed point to each coronary ostium. The elements of MDP are described in this section.

**Figure 2:**
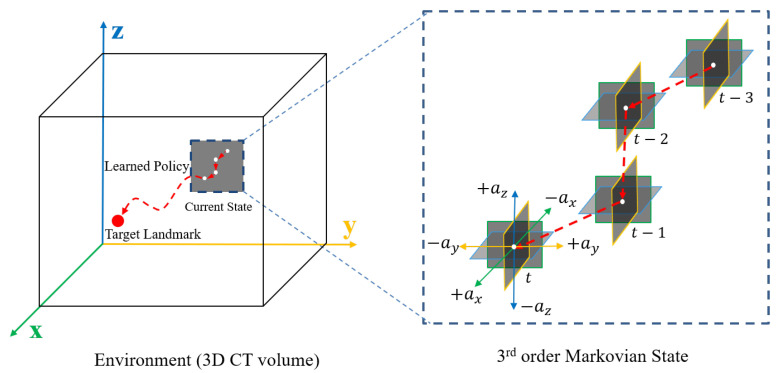
Schematic diagram of an environment: The CT volume is defined as the environment, and the trained agent aims to find the target location optimally. Local 2.5D patches at the current position *t* along the 3D trajectory are configured using the state of four histories $\left\{s_{t-h}|0\leq h\leq H\right\}$ such that the orientation can be learned together. *H* is the order of the Markovian property and H=3 in the example. The action set $A=\{\pm a_{x},\pm a_{y},\pm a_{z},0\}$ consists of six three-dimensional actions, including a stationary action.

**State:** In a 3D CCTA environment, we define state $s_{t}\in S$ as a 2.5D region of interest centered around the agent location for memory efficiency during training. Only three 2D patches ($3\times N^{2}$), the normal of which is the *x*-, *y*-, *z*-axis, respectively, are considered instead of considering the entire boxed 3D patch ($N^{3}$). Moreover, in the sequence of the search trajectory, the last three previous states are contextually considered. Considering them lets the agent know where the agent came from and will move to, stabilizing the search trajectories. Thus, the state used by the proposed method can be expressed as $s_{t}=\left\{s_{t-h}|0\leq h\leq H\right\}$, which has a *H* order of the Markovian property. We chose the third-order Markovian property (H=3), and the agent observes $H\times 3\times N^{2}$. The diagram of the state is shown in Figure 2.

**Action:** The action set *A* is composed of six 3D-directional actions and a stationary action, $A=\{\pm a_{x},\pm a_{y},\pm a_{z},0\}$. The agent moves from $p_{i}$ to $p_{i+1}$ with a 3D unit displacement vector $\vec{a}$ using Equation (4). Here, ρ is a variable step size, which we set initially to ρ=3voxels(≈1 mm).

(4) $$p_{i+1}=p_{i}+\rho \vec{a}$$

**Reward:** A scalar reward, which is distance-based feedback, is chosen to drive the behavior of the agent approaching the target location. $R_{i+1}=\left|\right|{\vec{p}}_{i}-{\vec{p}}_{GT}{\left|\right|}_{2}^{2}-\left|\right|{\vec{p}}_{i+1}-{\vec{p}}_{GT}{\left|\right|}_{2}^{2}$ where R∈R. As the agent approaches or moves away from the target, the difference between the distance to the target in the previous stage and the distance to the target in the current stage, i.e., the reward *R* is increased or decreased.

### 4.2. EsseNet: Explicit Spatio-Sequential Encoding Policy Network

For the local observation in 3D learning tasks, 3D contextual information can be observed by sampling 3D patches $\Phi_{i}$ from a volume image *I* given a voxel location $p_{i}$. The 3D patches provide more the surroundings information at the voxel location to be classified; however, the size will be $N^{3}$, which is computationally and memory expensive. In addition, it is difficult to accumulate a large amount of experience and replay buffer when implementing a DQN. Considering the sizes of the current, the next states $2\times N^{3}$, and sequential history size *H*, we need to store the experience instance $\left(s,a,r{,}^{\prime }s^{\prime }\right)$ with a size of at least $H\times 2\times N^{3}+2$, which is difficult to accumulate in an experience buffer.

To reduce the computational cost and required size of input data for 3D learning, a 2.5D CNN [6] using three orthogonal view aggregation is an alternative for our network in terms of RL implementation. In CT scans, the axial, coronal, and sagittal $\Phi_{h,j=1},\Phi_{h,j=2},\Phi_{h,j=3}$ will be the three representative views as 2D patches in CT scans [8,21], and we can then use 2.5D patches tghat have a size reduced from $H\times 2\times N^{3}$ to $H\times 2\times 3\times N^{2}$.

Let the functions of the convolutional layers with the parameters $\theta_{j}$ be $\xi (\Phi_{h,j};\theta_{j})$. Importantly, the parameters $\theta_{j}$ are shared with respect to historical sequence *h*, but are not shared with respect to the image axes *j*. Then, the output vectors $t_{h,j}$ by $\xi (\Phi_{h,j};\theta_{j})$ are encoded with respect to each axis *j* and sequence *h* as,

(5) $$t_{h,j}\leftarrow \xi \left(\Phi_{h,j};\theta_{j}\right)$$

We then concatenate the convolution outputs $t_{h,j}$ in Equation (5) from each plane as $T_{h}={⋃}_{j=1}^{3}t_{h,j}$. The concatenated 2.5D information now needs to be processed through local inference. Let the functions of the local inference with the parameters $\omega_{h}$ for each sequence be $\psi (T_{h};\omega_{h})$, and the inferenced output vectors ${\bar{T}}_{h}$ for three view aggregation are then found as follows:

(6) $${\bar{T}}_{h}\leftarrow \psi \left(T_{h};\omega_{h}\right).$$

**Figure 3:**
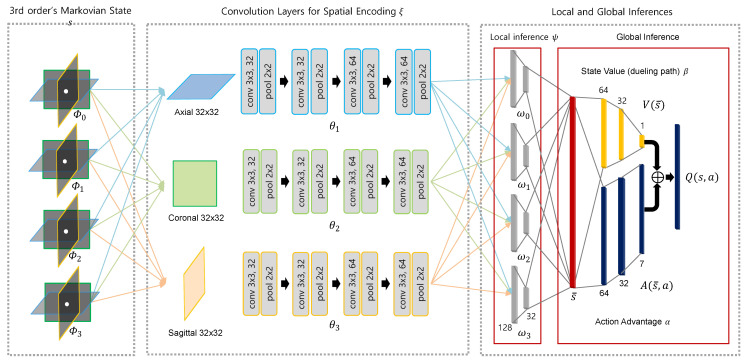
Explicit spatio-sequential encoding network (EsseNet): The four sequential states *s* and each orthogonal view patch Φ are independently encoded in the convolutional layer ψ. However, convolution parameters θ are shared for the same view despite being observed from different sequences. Features encoded from each view are collected into the corresponding sequence domain, and local inference is applied for each sequence. Finally, all information is concatenated, and the final action values can be obtained by passing through the dueling and action advantage paths.

Now, the input vector $\bar{s}$ of the global inference is found for both dueling and action advantage paths by $\bar{s}={⋃}_{h=0}^{H}{\bar{T}}_{h}$. The entire visualization of our process for explicit spatio-sequential encoding with several parameters from Equations (5) and (6) is described in detail in Figure 3.

A dueling DQN with our EsseNet is expressed as follows:

(7) $$Q\left(s,a;\Theta ,\Omega ,\alpha ,\beta \right)=V\left(\bar{s};\beta \right)+\left(A\left(\bar{s},a;\alpha \right)-\frac{1}{\left|A\right|}\sum_{a^{\prime }}A\left(\bar{s},a^{\prime };\alpha \right)\right)$$

where $\Theta =\{\theta_{j}|1\leq j\leq 3\}$ are the parameters of convolutional layers for the spatial encoding of the 2D planes $\Phi \left(s\right)=\left\{\Phi_{h}^{j}|0\leq h<H,\;1\leq j\leq 3\right\}$ with respect to the x-, y- and z-axes, $\Omega =\{\omega_{s}|0\leq h<H\}$ are the parameters of fully connected layers for sequential encoding of the aggregated 2D planes, and the global inference input vector $\bar{s}=\psi \left(\xi \left(\Phi \left(s\right);\Theta \right);\Omega \right)$. Here, α and β are the parameters for two streams of fully connected layers for the state value and action advantage, respectively.

Now, the 2.5D network is modeled for explicit spatial-sequential parameters *j* and *h*. EsseNet is not only accurate, but it also has significant efficiency in both memory and operation. The experience replay memory can be loaded approximately 10 times (N = 32) compared to the 3D patch-based network, increasing generality during the learning phase.

### 4.3. Localization Strategy for Robust Convergence

**Figure 4:**
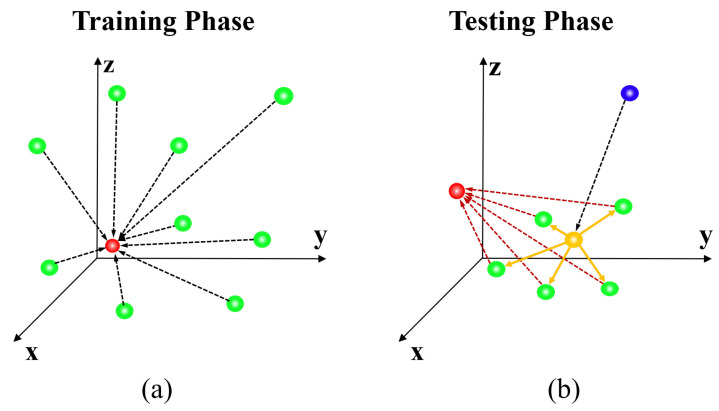
(**a**) Training phase: The agent directly tracks the target location (red) from randomly distributed locations (green). (**b**) Testing Phase: A Monte Carlo-like approach is used for better convergence. First, the agent moves from the center point (blue sphere) based on the learned policy with a fixed iteration. The agent converges with the predicted initial target (yellow sphere), which would be located near the target (red sphere), and new multiple seed points (green spheres) are generated using the random displacement vectors (yellow arrows) from the initial target. Now, the new agents quickly converge to the target (red sphere), and the final target location is defined by computing the weighted average of the multiple converged locations. Note that all dotted lines represent the trajectories based on the learned policy.

The agent reaches the target location from a random location by taking the displacement action step by step to maximize the reward considering the current state. It is challenging to set the termination criterion because it is impossible to specify a completely accurate location in the input data limited to a discrete space. Furthermore, because there is a trade-off between the convergence time and precision, the appropriate step size settings must also be considered. Owing to the property of the step size independence among historical states when using EsseNet, it is possible to apply the variable step size by decaying ρ in Equation (4) during the testing phase. In this paper, to reach the target location quickly and accurately, the agent takes a displacement action with a variable step size in a coarse-to-fine manner as described in Figure 4. First, the agent proceeds with a coarse step size of (ρ=3) based on the learned policy during a sufficient fixed number of steps (≈100 steps) from a random seed point, and the agent reaches the target location or somewhere near the target location. However, it is difficult to assure that its location is the true target location. The five new agents are then generated with a random displacement from the location of the first agent and proceed with a smaller step size (ρ=1) during a fixed number of steps (≈50 steps) to the target locations. Finally, we obtain a weighted average location from the five new agents for determining the final solution. The entire process is presented in Algorithm 1.
**Algorithm 1** The algorithm for Robust convergence
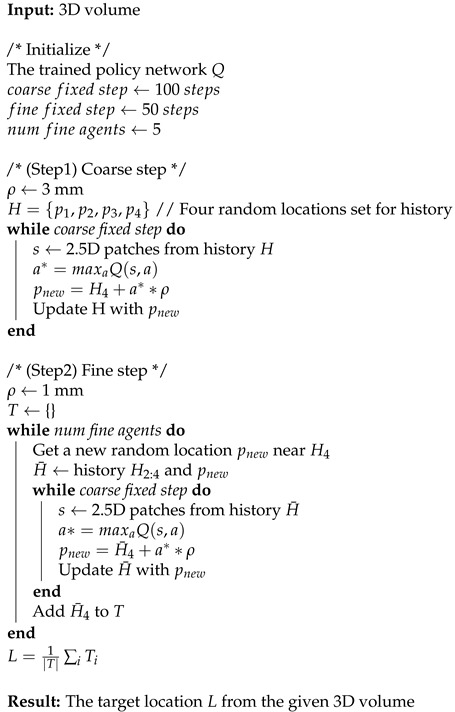



## 5. Experiments

The experiment was conducted using a publicly available dataset [26], consisting of 32 cardiac 3D-CT scans and the corresponding ground truth (GT). The dataset was intended for the extraction of a coronary artery centerline. Thus, the GT in the dataset was the trajectories from the coronary ostia to each distal end. Coronary ostia are important anatomical landmarks for the seed points of coronary artery segmentation and are included in our target landmarks. We trained the agents to find two ostia landmarks for LCA and RCA from 24 CT scans and tested using the remaining eight CT scans. As an evaluation metric, the accuracy based on the Euclidean distance error between the predicted and target points was measured for a quantitative comparison with the other state-of-the-art method. Furthermore, a comparison was made between EsseNet with DQN and EsseNet dueling DQN using average reward per episode.

### 5.1. Implementation Details

For the training phase, random seed points were sampled in 80% of the CT scans for the entire image dimension around the center. The training time for an agent took approximately 15 h using an NVIDIA TITAN X GPU. As parameters, we used a patch dimension of N=32, history order of H=3, and a batch size of 48. In addition, the experience replay memory size is $1.5\times e^{5}$. This amount of experience data can reside in a general desktop PC. Ten-times more experience instances can be loaded into the experience replay buffer than in other networks that use 3D patches for the state. The ADAM optimizer is used; the discount factor is set to γ=0.9; and ϵ is reduced from 0.9 to 0.1.

A single episode is completed and resets to the default parameters when the agent reaches the target location or moves outside the CT volume. Whenever a single episode is completed, the CT volume is randomly changed to maximize the quantity of the data. The agent moves using random actions when the network parameters are insufficiently optimized and similar experience data from a specific image case are then accumulated. To avoid such a situation, we limited the maximum number of moves to 1500 steps.

### 5.2. EsseNet with Dueling DQN

EsseNet, the network proposed in Section 4.2, is based on dueling DQN. We experimented with comparing the plain DQN [11] with dueling DQN [25] for stability and performance in identifying coronary ostia from 3D CT images.

Q-values from dueling DQN are separately modeled into the state value and action advantage, whereas Q-values from the plain DQN are directly inferred. Suppose the agent is located in a large anatomic part, such as an atrium, for which all image values are homogeneous. In that case, the agent attends the features regarding only the direction to the target from the aspect of the state value. The anatomic structures around the agent can sometimes be guides or obstacles for the agents. It is assumed that the action advantage enables the agent to attend to anatomic structures around the agent and choose actions for optimal shorter paths to the targets.

**Figure 5:**
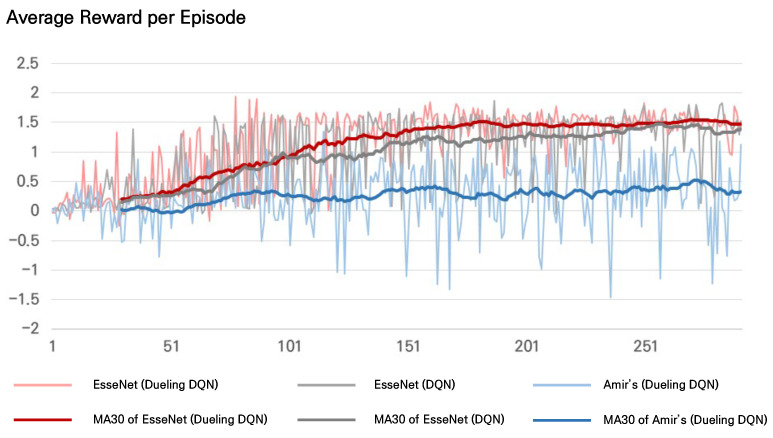
The average reward per episode during training: The average reward values per episode and their moving averages with a window size of 30 for the three methods, EsseNet with dueling-DQN (light red/dark red), EsseNet with plain DQN (light gray/dark gray), and Amir et al. [2] (light blue/dark blue) are presented, respectively.

The experiment was conducted by varying only the Q-approximator in the inference layer (plain DQN vs. dueling DQN) in EsseNet while the other encoding layers were fixed. Average rewards per episode were measured from both models to compare the learning trends. EsseNet with dueling DQN learned quickly with higher average rewards as shown in Figure 5. In addition, the accuracy was measured for a quantitative comparison in Table 1. EsseNet with dueling DQN showed better performance in both RCA and LCA.

### 5.3. Quantitative Evaluation and Comparison

We measured the accuracy based on the Euclidean distance error between the predicted and target locations for the comparison. Amir et al. [2] proposed DQN-based architectures that use a 3D patch with three sequential history buffers as the state inputs. They consider the history buffers to stabilize the search trajectories and prevent the agent from becoming stuck in repeated cycles. Because the proposed method also uses history buffers, Amir’s method is appropriate for comparison with the proposed method.

The most different parts between the proposed approach and Amir’s method were the structures of the input state and encoding layers. The 3D patches were encoded as input channels in Amir’s method, whereas each 2.5D patch was encoded explicitly from the proposed method.

As the results indicated, EsseNets with both plain DQN and dueling DQN showed a higher accuracy, as shown in Table 1. EsseNet achieved the best performance with dueling DQN.

We measured average rewards per episode during training from both EsseNet and Amir’s method. As shown in Figure 5, the trends of the average rewards per episode, the moving average with a window size of 30, showed that both methods with EsseNet were trained well. We found a significant difference between EsseNet and Amir’s method in the average reward per episode. EsseNet seemed to be stabilized with a slight variation of the average reward per episode after approximately 100 episodes. In contrast, the average rewards for Amir’s method were lower, and its variations were larger for all episodes.

Eight detailed CT images for the test cases and results by EsseNet with dueling DQN are shown in Table 2. The Euclidean distance errors are 2.22 ± 1.12 and 1.94 ± 0.83 to identify the ostia of LCA and RCA, respectively.

Table 3 shows the results from the literature for localizing the coronary ostia on different datasets except for Amir et al. [2]. The other methods used the local region of interest and the segmentation contours that contained the target landmarks as prior information. In contrast, the proposed method and Amir’s method did not use prior information, and the agents could not find the target landmarks from any randomly initialized location.

Regarding time analysis, it may depend on the computing power and the initial seed locations. Many deep learning-based investigations use floating-point operations (FLOPs), which is known to be one of the objective methods for analyzing the computational efficiency [27]. We also measured the number of FLOPs for the methods. There were about 2.4M and 2.5M FLOPs on both EsseNet (DQN) and EsseNet (Dueling DQN), respectively, while the 3D-based method [2] had 23.7M FLOPs. There was about a 10-fold difference in the computational efficiency between EsseNet and 3D-patch-based methods in our experiment.

**Figure 6:**
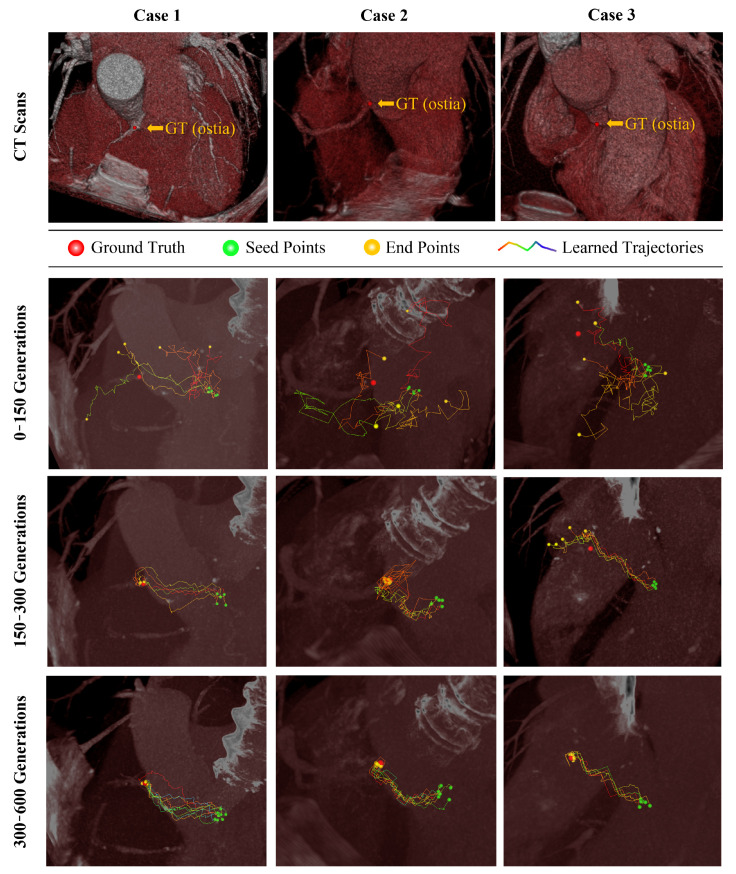
Visualization of training agents for coronary ostia. Robust convergence over a generation is observed for coronary ostia detection problems. GT, ground truth.

The search trajectories of the agent during training are shown in Figure 6. The trajectories from multiple seed points are unstable, so no agents identified the target landmarks after 150 episodes. Although some agents seemed to identify target landmarks after 300 episodes, others wander around or failed to identify the target landmarks. After 600 episodes, all agents directly searched and identified the target landmarks.

## 6. Discussion

The DRL-based methods for object detection were highly efficient and accurate, which is attractive for medical imaging applications. In this paper, we proposed a DRL-based efficient architecture considering 2.5D-patch images to identify coronary ostia in 3D CT images.

In most methods, only information observed around the agent at the current location was considered, and the Q value was then inferred based on the information. However, a single piece of ROI information around an agent’s current location may not be sufficient to determine an action when the number of possible states is huge such as in 3D medical images. In contrast, with the proposed method, multiple history buffers, i.e., the ROIs from previous locations, were considered together based on the previous trajectory, which made our state information include the properties of one small trajectory. These properties made it possible to consider where the agent came from and what action was optimal for the agent to go forward to the target object. This may have prevented the agent from staying around a specific location by creating repeated cycles [2,10,11,12]. Furthermore, a smoother and more stable trajectory was obtained overall.

Three-dimensional patches have more contextual information; however, when the history buffer is considered with it, the 3D volume patch can be a burden to accumulate in experience memory. Further, information is likely to overlap each other among the volume patches, leading to wasted memory. DQN-based networks learn by using the experience buffer as a data pool. The larger the size of a state, the more limited the data that accumulate in memory. To become more efficient and accumulate more information in the experience buffer, we considered the 2.5D patch-based architecture and designed EsseNet to encode the features of 2.5D patches efficiently. The use of a 2.5D patch avoids overlapping information as much as possible and allows for the accumulation of more data owing to its smaller size. EsseNet is not only accurate, but it also has significant efficiency in both memory and operation utilities and shows a higher performance than the other method using 3D patches.

The proposed method was affected more by the accuracy of the GT. The fact that the target locations defined by several experts had errors meant that determining the representative location of the target was difficult. In our dataset, the IO error ranged from a minimum of 0.26 to a maximum of 1.2 mm, with an average of 0.76 mm for the LCA and 0.55 mm for the RCA ostium. Reinforcement learning only trusts and learns the location of the GT; thus, there was a limit to producing a smaller error than the IO error. In addition, the surrounding structures of the target object should be consistent with all image data because these structures can be a guide for agents. Otherwise, learning the agents may be difficult, even if the IO error is small. For example, when the tip points of coronary arteries were set as targets, the locations and the surrounding structures for each type of data were not always consistent. In this case, it was more challenging to train the network.

## 7. Conclusions

In this paper, we proposed an efficient and accurate network, EsseNet, based on 2.5D patches and used in a DRL framework. It is designed to solve the sequential decision process problem in 3D CT images efficiently. A 2.5D patch-based state with three history buffers that have both spatial and sequential information is explicitly encoded. EsseNet is not only accurate, but it also has significant efficiency in both memory and operational utility and shows a higher performance than the other method using a 3D patch. As a result, the ostia of LCA and RCA achieved an accuracy of 1.94 ± 0.83 mm and 2.22 ± 1.12, respectively. Also, floating-point operations (FLOPs) were calculated for measuring the computational efficiency, and the result showed that there were 2.5M FLOPs on the proposed method. In comparison, the 3D-based method had 23.7M FLOPs. The proposed method can be integrated with other approaches to segment coronary arteries and applied to other identification tasks simply by changing the target locations in GT. We are conducting future investigations based on CT images and various image modalities, and large-scale data.

## Figures and Tables

**Table 1:** Comparison between 3D patch with history [2] and 2.5D patch with history (EsseNet); DQN, deep Q-learning; RCA, right coronary artery; LCA, left coronary artery.

Method	Error (mm)	
	RCA	LCA
Amir et al. [2] (dueling DQN)	5.67 ± 2.04	5.90 ± 1.95
EsseNet (DQN)	2.01 ± 0.86	2.47 ± 1.27
EsseNet (dueling DQN)	1.94 ± 0.83	2.22 ± 1.12

**Table 2:** The detailed test image cases with IO error and the results of identification of two coronary ostia by EsseNet with dueling DQN.; IO, inter-observer; DQN, deep Q-learning.

Test Dataset	Image Details and Measures (mm)				
	**Image Quality**	**Left Coronary Ostium**	**IO Error**	**Right Coronary Ostium**	**IO Error**
0	Moderate	4.50	0.53	3.66	0.26
1	Moderate	2.90	1.2	2.17	0.72
2	Good	1.56	0.42	1.08	0.35
3	Poor	1.52	1.05	1.28	0.5
4	Moderate	1.35	0.54	1.76	0.74
5	Poor	1.92	1.07	1.32	0.46
6	Good	2.79	0.79	2.39	0.43
7	Good	1.19	0.50	1.83	1.0
Average		2.22 ± 1.12	0.76 ± 0.30	1.94 ± 0.83	0.55 ± 0.24

**Table 3:** General comparison with the existing works for the localization of coronary ostia. It is difficult to compare the methods listed in the table directly since their results were reported on the different datasets used in the source papers. RTW [28] was introduced for 3D human pose estimation, but it is newly applied for localization of coronary ostia in the other work [29]. Hence, the result of RTW can be referred from the paper [29]. TAVI cases are the CT images scanned from patients who have cardiovascular diseases. Note that all the other methods use some prior information, while the proposed method and Amir’s method do not use any prior information.; RTW, random tree walk; TAVI, transcatheter aortic valve implantation.

Method	Mean Error (mm)	Approach	Priors	Data Type
Colonial walk [29]	2.01 ± 1.02	Random walk	Local region of interest	Private
Colonial walk [29]	2.05 ± 1.08	Random walk	Local region of interest	Private (TAVI)
RTW [28,29]	2.18 ± 1.25	Random walk	Local region of interest	Private
RTW [28,29]	2.44 ± 1.72	Random walk	Local region of interest	Private (TAVI)
Elattar [30]	2.18 ± 1.37	Image processing	Segmentation	Private
Amir et al. [2]	5.80 ± 1.99	Reinforcement learning	-	Public
EsseNet(dueling DQN)	2.08 ± 0.98	Reinforcement learning	-	Public

## Data Availability

Not applicable.
